# Epidemiological and Clinical Characteristics of Patients With Coronavirus Disease-2019 in Shiyan City, China

**DOI:** 10.3389/fcimb.2020.00284

**Published:** 2020-05-22

**Authors:** Long Liu, Xu Lei, Xiao Xiao, Jing Yang, Jian Li, Manshan Ji, Weixing Du, Huabing Tan, Jianyong Zhu, Bei Li, Zhixiong Jin, Weiyong Liu, Jianguo Wu, Zhixin Liu

**Affiliations:** ^1^Department of Infectious Diseases, Department of Respiratory, Renmin Hospital, School of Basic Medical Sciences, Hubei University of Medicine, Shiyan, China; ^2^Department of Clinical Laboratory, Tongji Hospital, Tongji Medical College, Huazhong University of Science and Technology, Wuhan, China; ^3^Guangdong Provincial Key Laboratory of Virology, Institute of Medical Microbiology, Jinan University, Guangzhou, China

**Keywords:** SARS-CoV-2, asymptomatic infections, coinfection, COVID-19, *Mycoplasma pneumonia*

## Abstract

To investigate the early epidemic of COVID-19, a total of 176 confirmed COVID-19 cases in Shiyan city, Hubei province, China were surveyed. Our data indicated that the rate of emergence of early confirmed COVID-19 cases in Hubei province outside Wuhan was dependent on migration population, and the second-generation of patients were family clusters originating from Wuhan travelers. Epidemiological investigation indicated that the reproductive number (R_0_) under containment strategies was 1.81, and asymptomatic SARS-CoV-2 carriers were contagious with a transmission rate of 10.7%. Among the 176 patients, 53 were admitted to the Renmin Hospital of Hubei University of Medicine. The clinical characteristics of these 53 patients were collected and compared based on a positive RT-PCR test and presence of pneumonia. Clinical data showed that 47.2% (25/53) of COVID-19 patients were co-infected with *Mycoplasma pneumoniae*, and COVID-19 patients coinfected with *M. pneumoniae* had a higher percentage of monocytes (*P* < 0.0044) and a lower neutrophils percentage (*P* < 0.0264). Therefore, it is important to assess the transmissibility of infected asymptomatic individuals for SARS-CoV-2 transmission; moreover, clinicians should be alert to the high incidence of co-infection with *M. pneumoniae* in COVID-19 patients.

## Introduction

By the end of 2019, just before the Chinese New Year, an outbreak of idiopathic pneumonia surfaced in Wuhan, China (Li Q. et al., 2020). Soon afterwards, the causative pathogen was identified as a novel coronavirus (Huang et al., 2020; Wang D. W. et al., 2020). With rapidly increasing clinical cases, person-to-person transmission was confirmed (Chan et al., 2020b). This novel coronavirus was later named by the International Committee on Taxonomy of Viruses as severe acute respiratory syndrome coronavirus 2 (SARS-CoV-2) (Coronaviridae Study Group of the International Committee on Taxonomy of Viruses, 2020). On January 30, 2020, a public health emergency of international concern was declared by the World Health Organization (WHO) (Public Health Emergency of International Concern declared by WHO, 2020). By March 30, 2020, a total of 82,545 coronavirus disease 2019 (COVID-19) cases were confirmed in China (China Center for Disease Control and Prevention, 2020) and more than 780,000 confirmed COVID-19 cases were identified globally (WHO Director-General's Opening Remarks at the Media Briefing on COVID-19-11 March, 2020). In Hubei Province, there were 67,801 confirmed cases (49,986 in Wuhan), including 7,984 severe cases (7,049 in Wuhan) and 3,187 deaths (2,430 in Wuhan) (China Center for Disease Control and Prevention, 2020). As previously reported, the most common symptoms at onset of COVID-19 were fever, cough, expectoration, headache, myalgia or fatigue, diarrhea, and hemoptysis, along with abnormal lesions on chest computed tomography (CT) (Shi et al., 2020; Wang C. et al., 2020; Wu and McGoogan, 2020). There was also evidence of lymphopenia in a proportion of patients (Chen H. J. et al., 2020).

The movement of millions of people with no effective protection measures is considered one of the main reasons for the spread of the epidemic; in particular, the massive population inflows from Wuhan to other hometowns before the Spring Festival fostered the outbreak of this disease to other regions. During the spread, interventional measures including lockdown of public places, cessation of highways and city traffic, wearing facial masks when outside, and refusion of social activities were taken to lower the transmissibility (Wuhan Novel Coronavirus Infection Pneumonia Epidemic Prevention Control Headquarters., 2020). Collection and analysis of the epidemiological and clinical characteristics of confirmed cases outside Wuhan helped to adopt and adapt strategies, resulting in prevention and control of the pandemic in these regions.

In this study, we summarized the dynamics and clinical features of the COVID-19 pandemic in Shiyan city in the Hubei province, a city 440 km from Wuhan city, based on the surveillance data up to February 24, 2020. It is very important to understand the epidemiological and clinical characteristics of COVID-19 in the surrounding cities of Wuhan. We hope that these data can provide positive suggestions for other cities to prevent the further spread of COVID-19.

## Materials and Methods

### Ethics

This study was conducted in accordance with the tenets of the Helsinki declaration. This study was approved by the institutional review board of Shiyan Renmin Hospital of Hubei University of Medicine, and the need for informed consent was waived. This study was designed as a retrospective case series, and no patients were directly involved in the study design, setting of research questions, or outcome measures. No patients were consulted for advice on interpretation or writing of results.

### Epidemical Data Sources

Data of the 176 confirmed COVID-19 patients were collected from January 22, 2020 to February 6, 2020, and included seven children aged <14 years and 169 adults. COVID-19 was confirmed by two positive RT-PCR tests in hospitals. Asymptomatic carriers were quarantined at the hospital or hotels after having been discovered.

Per the guidelines on investigation and management of close contacts for COVID-19 patients issued by the Chinese Center for Disease Control, close contacts of suspected cases, clinically diagnosed cases, and confirmed cases 2 days before the onset of illness were required to meet the following criteria: family members living in the same room, medical workers without secondary protection, and sharing personal meals or communication in confined spaces. The contact traces of confirmed cases were informed by patients or family members, and the duration spans 14 days before onset.

### Clinical Data Sources

Suspected cases were defined as meeting two of the following criteria: (1) fever, and/or respiratory symptoms; (2) presence of radiographic pneumonia; and (3) white blood cell (WBC) counts within upper limit of normal (ULN) or hypo-lymphocytosis during early course of the disease. Once the cases were identified, respiratory tract secretions and other samples were collected for real-time fluorescence reverse transcription-polymerase chain reaction (RT-PCR). In all, 176 patients who tested positive for SARS-CoV-2 nucleic acids were identified as confirmed cases and enrolled in the study.

Of these 176 confirmed patients, 53 (26 male and 27 female; mean age, 38 ± 17 years; age range, 6 months to 80 years) were admitted to the Department of Infectious Diseases, Department of Respiratory, Shiyan Renmin Hospital, Hubei University of Medicine. Data were collected and analyzed from the 53 patients from January 23, 2020 to February 24, 2020 (Table 1). The inclusion criteria were as follows: Suspected cases were screened according to the diagnosis and treatment protocol for COVID-19 pneumonia [Diagnosis and Treatment Protocol for Novel Coronavirus Pneumonia (6rd Interim Edition), 2020].

**Table 1:** Personal and clinical characteristics of 53 patients with COVID-19 in Shiyan city, Hubei province, China.

**Characteristics**	**All patients (*n* = 53)**
Median (interquartile) age (in years)	38 (28–47)
**Age groups (in years)**	
≤ 14	6 (11.3%)
15–30	12 (22.6%)
31–59	29 (54.7%)
≥60	6 (11.3%)
**Sex**	
Male	26 (49.0%)
Female	27 (50.9%)
**Coexisting infection**	
*Mycoplasma pneumoniae*	25 (47.2%)
Other pathogens	6 (11.3%)
**Coexisting conditions**	
Any	26 (49.0%)
Hypertension	3 (5.7%)
Diabetes	1 (1.9%)
Chronic obstructive pulmonary disease	8 (15.1%)
Cerebrovascular disease	3 (5.7%)
Renal disease	3 (5.7%)
Liver disease	9 (17.0%)
**Exposure history in Wuhan** ** <2 weeks**	
Yes	6 (11.3%)
No	47 (88.7%)
Familial cluster	32 (60.4%)
Fever	46 (86.8%)
**Highest temperature (°C)**	
<37.3	7 (13.2%)
37.3–38.0	12 (22.6%)
38.01–39.0	24 (45.3%)
>39.0	10 (18.9%)
Cough	35 (66%)
Myalgia or fatigue	17 (32.1%)
Expectoration	32 (60.4%)
Hemoptysis	1 (1.9%)
Headache	14 (26.4%)
Diarrhea	3 (5.7%)

*Values are medians (interquartile range) or numbers (percentage)*.

### Sample Collection and Pathogen Identification

After admission to Shiyan Renmin Hospital, indirect immunofluorescent assay was performed to simultaneously detect IgM antibodies against the following main etiological agents of pneumonia: *Legionella pneumophila, Mycoplasma pneumoniae, Coxiella burnetii, Chlamydia pneumoniae*, adenovirus (AdV), influenza A virus (IAV), influenza B virus (IBV), parainfluenza virus type 1+2+3 (PIV 1+2+3), and respiratory syncytial virus (RSV). The Pneumoslide-M kit (Vircell IFA KIT) was used for testing according to the manufacturer's instructions. IgM antibody detections for *Mycoplasma pneumoniae* of the coinfection pneumonia patients were performed at least three times during the acute phase and recovery phase. IgM antibody for *Mycoplasma pneumoniae* was also quantified by Serodia-Myco II assay (Fujirebio Inc., Tokyo, Japan), and IgG antibody were tested by the mycoplasma EIA kit (EUROIMMUN Inc., German).

In addition, respiratory tract samples including sputum and nasopharyngeal swabs collected from the patients were tested for severe acute respiratory syndrome-associated coronavirus (SARS-CoV) and Middle East respiratory syndrome coronavirus (MERS-CoV) by using Ag Path-ID One-Step RT-PCR Kit (Cat: AM1005, ABI) according to the manufacturer's instructions.

Respiratory tract samples were also used for real-time fluorescence RT-PCR to detect the presence of SARS-CoV-2 by using the SARS-CoV-2 (ORF1ab/N) nucleic acid detection kit (Cat: SJ-HX-009-2, Bio-germ, Shanghai, China) according to the manufacturer's instructions.

### Antiviral Treatment

Interferon alpha (5 million U or equivalent dose per time for adults, 2 times a day for atomization inhalation), lopinavir (200 mg/pill for adults, 2 pills for each time, 2 times a day, the course of treatment was <10 days), ritonavir (50 mg/pill for adults, 2 pills for each time, 2 times a day, the course of treatment was <10 days), ribavirin (500 mg/pill for adults, 2–3 times a day for intravenous infusion, the course of treatment is not more than 10 days), and Abidol (200 mg for adults, 3 times a day, the course of treatment was not exceed 10 days) were used. Antiviral traditional Chinese medicine was used for adjuvant treatment.

### Clinical Data Collection

Basic demographic and clinical data including age, sex, underlying diseases, and comorbidities were collected for each patient (Table 1). Laboratory findings of COVID-19 patients categorized by *M. pneumoniae* lgM antibody presence were recorded (Table 2). In addition, epidemiological histories were taken. Laboratory test results of standard blood counts (absolute white blood cells and lymphocytes); blood biochemistry (alanine transaminase, aspartate transaminase, creatine kinase, and creatinine); coagulation function; procalcitonin; C-reactive protein; erythrocyte sedimentation rate; and myocardial enzyme spectrum were compiled (Table 3). Additional data collected included medical imaging; treatment regimens (antiviral, antibacterial, systemic corticosteroid, immunoglobulin G, respiratory support); and prognosis (recovered and discharged, inpatient treatment, or death) (Table 4).

**Table 2:** Laboratory findings of COVID-19 patients categorized by *M. pneumoniae* lgM antibody presence.

	***Mycoplasma*** **lgM (–)**	***Mycoplasma*** **lgM (+)**	***P*-value**
Neutrophils (%)	70.28 ± 2.558	59.64 ± 3.119	0.0264^*^
Lymphocytes (%)	27.82 ± 3.389	34.41 ± 5.348	0.2904
Monocytes (%)	9.733 ± 1.615	18.18 ± 1.654	0.0044^**^
White blood cells (× 10^9^/L)	4.442 ± 0.399	5.046 ± 0.455	0.3242
CRP (mg/L)	15.04 ± 5.471	13.09 ± 4.005	0.7787
LDH (U/L)	254 ± 43.50	272 ± 57.25	0.8435

*, ** *means a significant difference*.

**Table 3:** Laboratory findings in patients with COVID-19.

**Variables**	**All patients (*n* = 53)**
White blood cell count (× 10^9^/L)	4.68 (3.32**–**5.08)
<4	21 (39.6%)
4–10	32 (60.4%)
Neutrophil count (× 10^9^/L)	2.76 (1.96**–**3.81)
**Lymphocyte count (× 10**^**9**^**/L)**	
<1.0	22 (41.5%)
≥1.0	31 (58.5%)
Hemoglobin (g/L)	123.0 (116.9**–**148.2)
**Platelet count (× 10**^**9**^**/L)**	
<100	2 (3.8%)
≥100	51 (96.2%)
**C-reactive protein (mg/L)**	
<5	31 (58.5%)
≥5	22 (41.5%)
**Aspartate aminotransferase (U/L)**	
<40	42 (79.2%)
≥40	11 (20.8%)
Potassium (mmol/L)	3.2 (2.9**–**3.6)
Sodium (mmol/L)	136 (128**–**142)
**Creatinine (μmol/L)**	
≤ 133	51 (96.2%)
>133	2 (3.8%)
**Creatine kinase (U/L)**	
≤ 185	48 (90.6%)
>185	7 (13.2%)
**Lactate dehydrogenase (U/L)**	
≤ 245	46 (86.8%)
>245	7 (13.2%)
**Procalcitonin (ng/mL)**	
<0.1	42 (79.2%)
≥0.1	11 (20.8%)
Pneumonia	53 (100%)

*Values are medians (interquartile range) or numbers (percentage)*.

**Table 4:** Treatment regimen and prognosis of patients with COVID-19.

**Treatment**	***n* (percentage)**
Antiviral	53 (100%)
Antibacterial	25 (47.2%)
Systemic corticosteroid	12 (22.6%)
Human γ-immunoglobulin	12 (22.6%)
Respiratory support	48 (90.5%)
Nasal cannula	12 (22.6%)
Non-invasive ventilation	32 (60.4%)
Improved and discharged	53 (100%)
Inpatient treatment	53 (100%)
Death	0 (0%)

*Values are numbers (percentage)*.

### Statistical Analysis

Epidemiological and clinical data were collected and analyzed by Microsoft Office (version 2016) and GraphPad Prism (version 5.0), and the epidemiological figures were plotted using Microsoft Excel. Continuous clinical data were expressed as medians and ranges, and categorical data, as counts and percentages.

## Results

### Dynamics of the COVID-19 Epidemiology in Shiyan City

**Figure 1:**
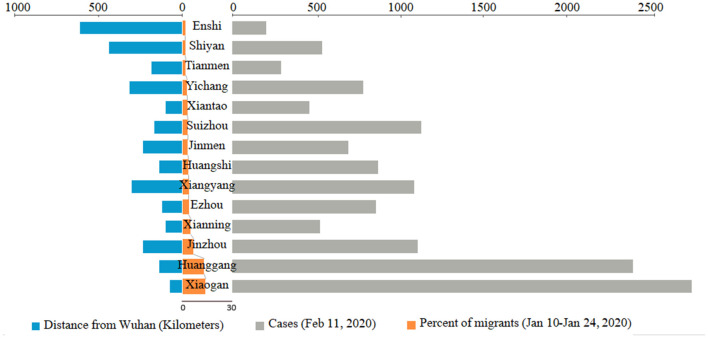
The population migration and confirmed COVID-19 cases in Hubei province outside Wuhan. Flow of population migration from Wuhan to other cities in Hubei province between January 10 and January 24, 2020, during the “Chunyun” period. Data of COVID-19 cases were collected from the Chinacdc.com.

**Figure 2:**
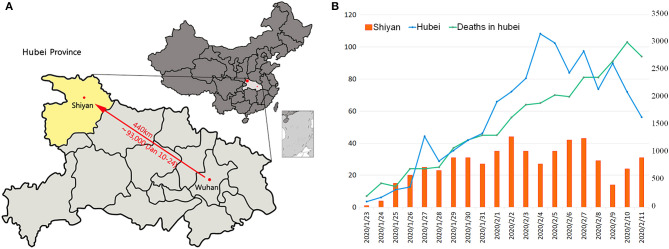
Analysis of the population migration and trend of illness onset. **(A)** Geographical display of the distance of Shiyan from Wuhan. The migrant population is calculated using the percent of total migrated individuals. **(B)** The onset numbers of confirmed COVID-19 patients in Hubei province and Shiyan city. Deaths occurred up to February 11, 2020 in Hubei province were also counted.

The resident population of Wuhan, the capital of Hubei province, was 11.081 million at the end of 2018, and the migrant population exceeded 5 million (National Health Commission of the PRC, 2020). At the beginning of “Chunyun” (migration during Spring Festival) from January 10, 2020 to January 24, 2020, most of the migrants from Wuhan travel to other counties and cities in Hubei province, accounting for about 69.4% of the total migrating population. They travel especially to Xiaogan (13.8%) and Huanggang (13.04%) (Data Came from Baidu Qianxi Map, 2020), which are adjacent to Wuhan (Figure 1). As of midnight of February 11, 2020, the confirmed COVID-19 cases in the cities outside Wuhan are migrate rate-dependent emerged (Figure 1). In Shiyan city, located in the southwest of Hubei province and 440 km from Wuhan city, the migrant population was 1.86% (about 93,000 people) which accounted for people who came back from Wuhan in the period between January 10 and January 24, 2020 (Figure 2A). Based on the incubation period of SARS-CoV-2, the epidemiological data of confirmed COVID-19 cases that emerged in Shiyan were collected from January 23, 2020. The number of confirmed COVID-19 cases onset in Hubei province showed a rapid increase before February 4, 2020, peaking at 3,156, and then showed a gradually decreasing trend (Figure 2B). The same trend of newly confirmed cases was also found in Shiyan city, with a peak of 44 cases that fluctuated between February 2 and February 7, 2020. As of midnight of February 23, there were a total of 669 confirmed cases in Shiyan, and only two related deaths. However, 374 patients were still under treatment in hospital including 20 with severe illness and 15 with critically severe illness. The overall case fatality rate (CFR) of COVID-19 patients in Hubei province was 3.82% from January 23 to February 11, 2020.

**Figure 3:**
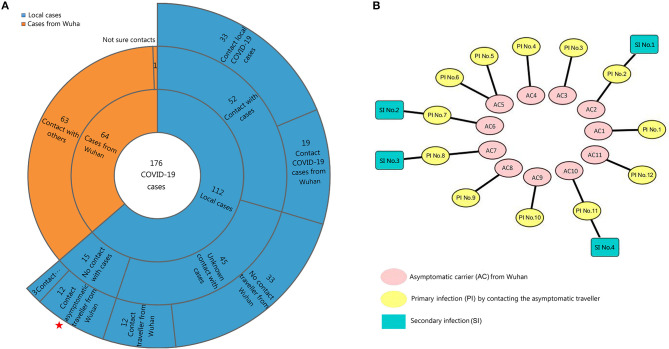
Contact history analysis of the 176 confirmed cases. **(A)** The contact history was obtained by patients or family members, and the duration spanned 14 days before symptom onset. Stars indicate the 12 cases after contact with infected asymptomatic carriers. **(B)** Twelve patients (primary infection, PI) infected by asymptomatic carriers (AC) from Wuhan; the secondary infections (SI) were surveyed. Eleven asymptomatic infections caused 12 primary infections and four secondary infections.

The 64 confirmed cases returning from Wuhan were surveyed. The 112 confirmed cases in local clusters without travel history to Wuhan implied that second-generation patients appeared in Shiyan through close contact; these included 52 local cases that had clear contact history with the COVID-19 patients from Wuhan or local citizens (Figure 3A). Fifteen of them with no close contact with COVID-19 patients, and 45 local cases with unknown sources had been infected with the virus. Notably, the onset of 12 cases were confirmed after close contact with 11 travelers from Wuhan who were asymptomatic carriers and showed no signs of illness after returning from Wuhan after nearly 1 month (Figure 3B). Another 12 cases caused four secondary infections in this period. The transmission rate caused by asymptomatic carriers was 10.7% (12/112).

**Figure 4:**
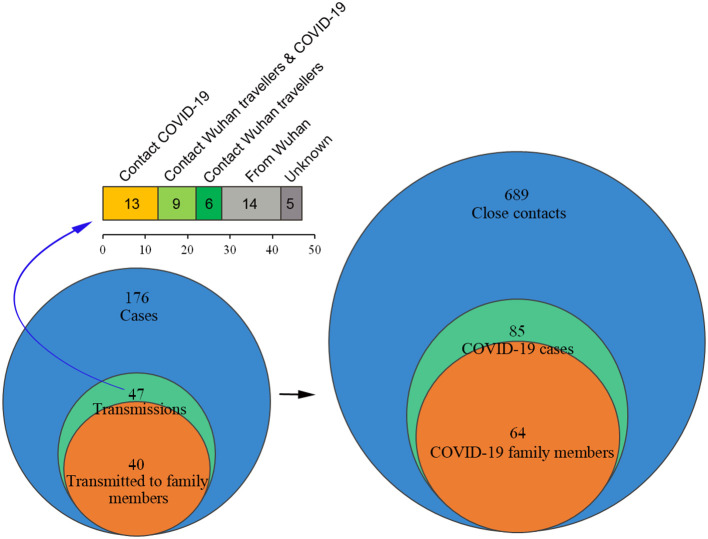
Survey of the close contacts of 176 confirmed cases. The close contacts mainly included family members, colleagues, or friends who lived together, shared meals, and/or physically communicated with the confirmed COVID-19 patients 2 days before the onset of illness. The close contacts were interviewed.

Among the 176 surveyed cases including Wuhan travelers and local citizens, 689 related close contacts were tracked (Figure 4). Forty-seven patients transmitted the virus and caused 85 confirmed cases, including 40 patients who transmitted the virus to 64 family members. The infection rate in our surveyed data was 12.34% (85/689), and the R_0_ was 1.81 (85/47), which is lower than the recent reports because of the family quarantine measures (Chen T. M. et al., 2020). Contact tracing of the 47 cases showed that 14 of them traveled back from Wuhan, 13 contacted COVID-19 patients, and nine came into contact with Wuhan travelers and confirmed COVID-19 cases. In addition, six patients had contact history with Wuhan travelers, and five showed unknown infection routes, which included three patients that transmitted the virus to their family members and colleagues and another two that spread the virus to their colleagues or friends.

### Clinical Characteristics of COVID-19 Cases in Shiyan

To better understand the clinical features of COVID-19 cases we tracked, up to February 23, 2020, the clinical data on 53 patients (26 male and 27 female) were collected in the Department of Infectious Diseases of Shiyan Renmin Hospital, Hubei Province, with laboratory-confirmed SARS-CoV-2 infection. Six (11.3%) of these patients were <14 years old, 12 (22.6%) were aged between 15 and 30 years, 29 (54.7%) were aged between 31 and 59 years, and six (11.3%) were ≥60 years. The median age was 38 years (interquartile range, 28–47 years) (Table 1). Interestingly, we noticed that 25 (47.2%) patients were co-infected with *M. pneumoniae* (Table 5), who had a lower neutrophils percentage (59.64 ± 3.119 vs. 70.28 ± 2.558, *P* < 0.0264) and higher monocytes percentage (18.18 ± 1.654 vs. 9.733 ± 1.615, *P* < 0.0044) compared with *M. pneumoniae* negative patients (Table 2). Six (11.3%) of the 53 COVID-19 patients were co-infected with other common respiratory pathogens, such as IAV, IBV, and RSV, respectively. Among the 53 COVID-19 patients, 26 (49.0%) had the following underlying diseases: three (5.7%) had hypertension, one (8%) had diabetes, eight (15.1%) had chronic obstructive pulmonary disease, three (5.7%) had cerebrovascular disease, three (5.7%) had renal disease, and nine (17.0%) had liver disease. Only six (11.3%) of the 53 patients had history of exposure in Wuhan. Twenty-two (60.4%) of the 53 patients were associated with familial clusters. The most common symptoms at illness onset were fever (46, 86.8%); cough (35, 66%); and expectoration (32, 60.4%). Other symptoms at illness onset were myalgia or fatigue (17, 32.1%); hemoptysis (1, 1.9%); headache (14, 26.4%); and diarrhea (3, 5.7%) (Table 1).

**Table 5:** IgM antibody titers for the *M. pneumoniae* co-infection patients.

**IgM antibody titer**	**Number (*n* = 25)**
1:40	1 (4%)
1:80	8 (32%)
1:160	9 (36%)
1:320	5 (20%)
1:640	2 (8%)

**Figure 5:**
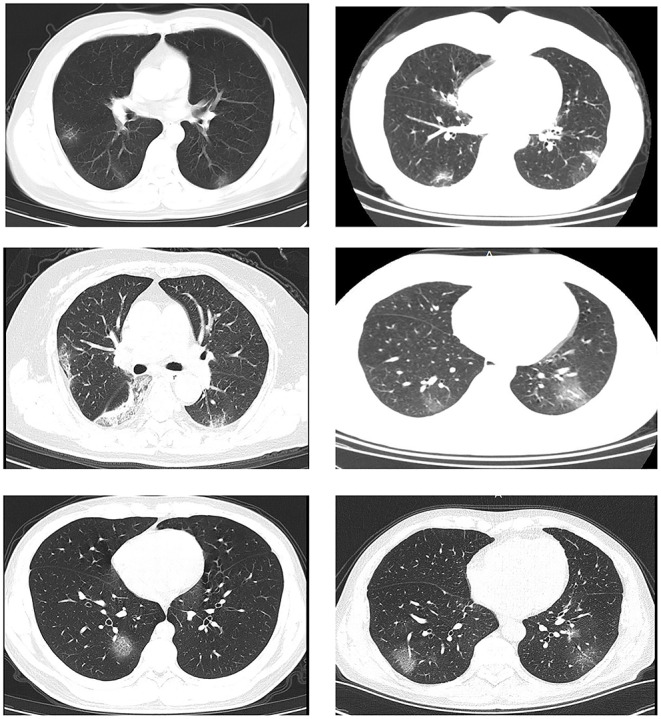
Transverse chest computed tomography images of patients with COVID-19. Transverse chest computed tomography of six patients with COVID-19 on admission showed bilateral or multiple lobular or subsegmental areas of consolidation or bilateral ground glass opacity.

On admission, the blood counts of 21 (39.6%) of the 53 patients showed leucopenia (white blood cell count: <4 × 10^9^/L) and 22 (41.5%) showed lymphopenia (lymphocyte count: <1.0 × 10^9^/L) (Table 3). The levels of C-reactive protein (CRP) were elevated in 22 (41.5%) patients and the levels of aspartate aminotransferase (AST) were increased in 11 (20.8%) patients. Twenty-two (79.2%) patients had normal serum levels of procalcitonin (PCT) (<0.1 ng/mL). All patients had pneumonia and showed abnormalities on either chest CT or radiographs. Typical chest CT findings of infected patients on admission were bilateral or multiple lobular or subsegmental areas of consolidation or bilateral ground glass opacity (Figure 5). Of the 53 patients, only one patient (age, 40 years) was transferred to an intensive care unit for acute respiratory distress syndrome and received mechanical ventilation.

All patients received antiviral treatment (Table 4). Among the 53 COVID-19 patients co-infected with *M. pneumoniae*, 25 (25/53, 47.2%) were given antibiotic (levofloxacin) treatment, and 12 (12/53, 22.6%) were given systematic corticosteroid and γ-immunoglobulin treatment. At the time of writing this paper, all 53 (100%) patients were discharged and there were no deaths. Fitness for discharge was based on subsiding of fever for at least 3 days, with improved evidence on chest radiography and viral clearance in samples from the lower respiratory tract.

## Discussion

In Shiyan city, the first laboratory-confirmed case of COVID-19 was identified in January 23, 2020, and the epidemic has experienced an increasing trend before February 2. The growth phase of new cases is consistent with most other regions outside Wuhan in Hubei province. Because CT-based diagnosis of COVID-19 was considered a confirmatory criterion in Hubei province [Diagnosis Treatment Protocol for Novel Coronavirus Pneumonia (Trial Version 6, Revised), WHO China Office, 2020], more than 10,000 patients were treated in hospital on February 12, 2020 (Lu J. et al., 2020). The growth trend in Hubei province and Shiyan city was lower than the expected growth curve because of the strict quarantine measures (Peng et al., 2020; Roosa et al., 2020). During the Chinese traditional new year, most family members and relatives gather at home and share the festivities. Therefore, cluster cases occurred mainly among family members and originated either from Wuhan travelers or those that came into contacting with COVID-19 patients. The origin of the virus could not be confirmed in five patients, and 12 patients had made contact with passengers traveling from Wuhan who were asymptomatic carrier and not in the incubation period. Although asymptomatic and presymptomatic infection of SARS-CoV-2 has been reported recently, most of them subsequently developed symptoms (Arons et al., 2020; Bai et al., 2020; Rothe et al., 2020). There was no evidence that these 11 individuals from Wuhan had an incubation period of more than 1 month and transferred the virus to their family members during this period. Currently published research basically reported that confirmed cases in the presymptomatic stage can result in transmission (Liu et al., 2020; Yu et al., 2020), it is yet not clear the transmissibility and transmission rate by asymptomatic carriers. Therefore, it is necessary to assess the transmissibility of asymptomatic carriers, strengthening the management of asymptomatic patients and tracing the close contacts of asymptomatic individuals can close the loophole.

According to the statistics of clinical cases, children seem to be less infected. The infection is mainly concentrated in the age group of 31–59 years. Children and youth have been less infected, which may be due to other unknown reasons. According to previous reports, men are more likely to be infected with SARS-CoV-2 than women (Yang et al., 2020), but our study found no significant difference in the infection rate of men and women. Most importantly, 25 (47.2%) of the 53 COVID-19 patients were co-infected with *M. pneumoniae*. Common respiratory pathogens such as seasonal influenza viruses were not common in the 53 COVID-19 patients. Because monocytes increased after *M. pneumoniae* infection alone (Puljiz et al., 2006; Wang et al., 2019), indicating the involvement of monocyte-related mechanisms in the pathogenesis of *M. pneumoniae* co-infection in COVID-19 patients. This suggests that we should pay more attention to *M. pneumoniae* co-infection for COVID-19 patients during clinical testing and corresponding treatment. The existence of underlying diseases may promote the generation of SARS-CoV-2 infection to a certain extent. This is also one of the reasons for the higher mortality rate of the elderly COVID-19 patients (Ji et al., 2020). Only a few patients had been to Wuhan, while most of the other patients acquired local infections. This confirmed the strong infectivity of SARS-CoV-2; therefore, controlling local clusters is key to prevent outbreaks from imported cases. Fever, cough, and expectoration are the main clinical symptoms of COVID-19. However, it is particularly interesting to note that 13.2% patients in our study showed no fever symptom despite being infected. This suggests that the severity of SARS-CoV-2 infection could be graded by combining CRP levels with the patient's age (Lu H. et al., 2020). In this study, we showed that 41.5% of patients had abnormally high CRP levels (≥5 mg/L). On admission, decreased leukocyte and lymphocyte counts indicated that the immune function of patients was compromised, consistent with a previous report by Xu et al. (2020).

From the perspective of clinical treatment, antiviral treatment (including antiviral traditional Chinese medicine) played a better therapeutic role. In addition, early detection of infection and symptomatic treatment were essential to reduce mortality. However, RT-PCR test results had a false-negative rate (Chan et al., 2020a; Li Z. et al., 2020). At present, asymptomatic carriers have been identified (Guan et al., 2020), and patients discharged from hospitals may still be carriers of the virus (Lan et al., 2020). Therefore, it is very important to find more effective detection methods. According to our clinical observation, CT imaging can effectively detect SARS-CoV-2 infection. However, fever, cough, and other related symptoms cannot be used as absolute evidence of infection.

Controlling and stopping the outbreak of a new pathogen that is effectively transmitted from person to person remains extremely challenging for most countries, especially when the SARS-CoV-2 has become a global pandemic having spread to 114 countries (WHO Director-General's Opening Remarks at the Media Briefing on COVID-19-11 March, 2020). Therefore, vaccine research is crucial for effective treatment and control of viral transmission; however, animal experiments and clinical trials are time consuming and cannot produce immediate results. China has heavily invested in medical resources to treat the COVID-19 patients, especially elderly patients with severe and critical illness. Strict intervention measures adopted by the government should be referenced in other regions with heavy outbreaks. Moreover, high-quality epidemiological investigations can find close contacts and early asymptomatic infections, reducing the potential risk of transmission by asymptomatic infection could lead to the stabilized epidemic.

## Data Availability Statement

All datasets presented in this study are included in the article/supplementary material.

## Ethics Statement

This study was approved by the Shiyan Renmin Hospital of Hubei University of Medicine institutional review board and the need for informed consent was waived.

## Author Contributions

LL, WL, JW, and ZL contributed to the design of experiments. LL, XL, XX, JY, JL, MJ, WD, HT, JZ, BL, and ZJ contributed to the conduction of experiments. LL, XL, XX, JY, WL, JW, and ZL contributed to the reagents. LL, XL, XX, JY, JL, MJ, WD, HT, JZ, BL, ZJ, WL, JW, and ZL contributed to the analyses of the data. LL, JW, and ZL contributed to the writing the paper. JW and ZL contributed to the editing the paper.

## Conflict of Interest

The authors declare that the research was conducted in the absence of any commercial or financial relationships that could be construed as a potential conflict of interest.
